# The Intersection of Heart Failure and Iron Deficiency Anemia: Diagnostic and Therapeutic Approaches

**DOI:** 10.2174/011573403X331380241111091452

**Published:** 2025-01-03

**Authors:** Maleesha Bw Thammitage, Naji Moussa, Ali Rezvani, Damandeep Kaur Dhillon, Miryam Lisseth Obando Gamarra, Kanwaraj Singh, Abdul Hawwa, Alejandra Felix Vincente, Sehajpreet Kaur, Kiranmayee Seshasai Nemalapuri, Devika Poonwassie, Manju Rai

**Affiliations:** 1 Aloka Medical Center, Horona, Ratnapura, Sri Lanka;; 2 Department of Cardiology, Richmond Gabriel University, Kingstown, St. Vincent, Caribbean;; 3 School of Medicine, Case Western Reserve University, Cleveland, OH 44106, USA;; 4 Saint James School of Medicine, Dallas, TX 75238, USA;; 5 Universidad Andina del Cusco, Cusco, Peru;; 6 Maharishi Markendeshwar Institute of Medical Sciences and Research, Ambala, Haryana, India;; 7 School of Medicine, St. George’s University, Grenada, West Indies;; 8 Department of Internal Medicine, Universidad de Sonora, Sonora, Mexico;; 9 Punjab Institute of Medical Sciences, Jalandhar, Punjab, India;; 10 Massachusetts College of Pharmacy and Health Sciences, Boston, MA 02115, USA;; 11 Department of Internel Medicine, University of West Indies, St. Augustine, Trinidad and Tobago;; 12 Department of Medical Biotechnology, Shri Venkateshwara University, Gajraula, Ludhiana, Uttar Pradesh, India

**Keywords:** Heart failure, iron deficiency anemia, prognosis, clinical outcomes, intravenous iron therapy, individualized approach

## Abstract

Iron deficiency anemia (IDA) is highly prevalent among individuals with heart failure (HF), impacting 40-70% of patients and serving as a significant prognostic indicator. Linked with oxidative metabolism and myocardial cell damage, IDA exacerbates HF symptoms, including reduced exercise capacity, diminished quality of life, and heightened cardiovascular morbidity. This review explores the diagnosis, treatment, clinical outcomes, prognostic indicators, and forthcoming challenges associated with IDA in HF patients. Crucially, addressing IDA in HF is critical for enhancing prognosis, including clinical outcomes, quality of life, hospitalizations, and survival rates. While oral iron therapy shows efficacy in reducing mortality and hospitalizations, it falls short in improving exercise capacity and quality of life, often deterring patients due to side effects. In contrast, intravenous (IV) iron therapy is highly effective in enhancing hematological parameters, functional capacity, and reducing HF hospitalizations. Optimizing IV iron dosing based on individual patient characteristics is essential for balancing treatment efficacy and adverse effects. Emphasizing individualized approaches, with IV iron emerging as a superior option, underscores the necessity for ongoing research to refine dosing strategies and explore novel therapies. Compliance remains paramount for positive outcomes with IDA treatment, with oral supplementation being cost-effective and easily accessible. However, parenteral supplementation proves beneficial for patients intolerant to oral therapy. Addressing IDA through tailored interventions, including oral or parenteral supplementation, is pivotal in averting complications and improving outcomes in HF patients. This paper consolidates insights into the diagnosis, treatment, impact, pathophysiology, clinical outcomes, research gaps, and future directions concerning IDA in HF patients, drawing on extensive literature to offer a comprehensive understanding of this critical issue.

## 1 INTRODUCTION

Iron deficiency anemia (IDA) impacts 30-50% of individuals with chronic heart failure [1]. Iron deficiency is prevalent in up to 80% of patients experiencing acute heart failure [2, 3]. The World Health Organization (WHO) defines anemia as hemoglobin levels below 13.0 g/dL in adult males and below 12.0 g/dL in adult females. Iron deficiency is generally defined as ferritin levels below 100 μg/L or ferritin levels ranging from 100 to 299 μg/L along with transferrin saturation (TSAT) below 20% [4]. IDA is characterized by reduced hemoglobin levels in red blood cells and decreased mean corpuscular volume, both resulting from low iron levels. IDA can be either absolute or functional. Absolute IDA occurs in the presence of depleted iron stores and occurs secondary to decreased intake, impaired absorption, increased demand, or chronic blood loss. Functional IDA refers to the hampered mobilization of stored iron secondary to chronic inflammation and increased hepcidin levels, as seen in patients with chronic conditions such as cancer and chronic kidney disease [3, 5, 6]. The primary consequence of iron deficiency is reduced erythropoiesis, leading to diminished oxygen-carrying capacity in the blood [7, 8]. This reduced oxygen availability particularly affects cardiac myocytes, which have high energy demands and are highly dependent on an adequate iron supply for proper function [1, 9].

Heart failure (HF) is a clinical syndrome characterized by the heart's inability to maintain adequate blood flow to meet the body's metabolic needs or to accommodate systemic venous return [10, 11]. It is associated with generalized inflammation, marked by increased immune response and elevated levels of pro-inflammatory mediators in the failing myocardium [12]. IDA is frequently linked to HF and typically correlates with more severe symptoms and poorer outcomes [13-15]. Furthermore, anemia leads to reduced exercise capacity and is linked with decreased survival [2, 16].

It has been shown that correction of iron deficiency by oral or intravenous (IV) iron supplementation improved well-being, and exercise tolerance and suggested reduced hospitalization and mortality [15, 17]. Therefore, the importance of understanding, diagnosing, and treating IDA can be seen in reducing its consequences on HF and improving outcomes. This article aims to review IDA, its pathophysiology, diagnosis, and treatment. The review analyzes clinical trials and current literature on diagnosing and treating IDA as well as looking at clinical outcomes. Additionally, the study aims to identify research gaps in the optimal treatment of IDA in HF.

## 2 PATHOPHYSIOLOGY

**Fig. (1):**
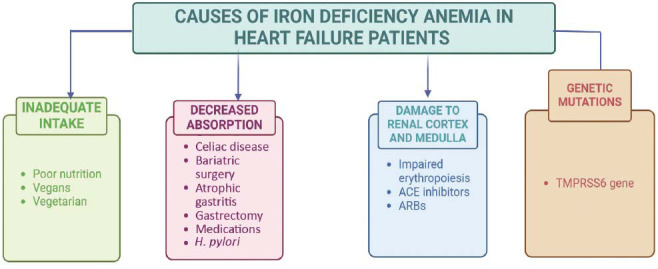
Causes of iron deficiency anemia (IDA).

Iron deficiency in HF is defined by International Guidelines as ferritin <100 ng/ml or ferritin 100-299 ng/ml with TSAT <20% [18, 19]. Its pathophysiology is multifactorial (Fig. **1**) and categorized as follows:

### 2.1 Reduced Iron Intake

Several studies indicate that malnutrition affects a significant proportion of HF patients, ranging from 35% to 78% [20-22]. Moreover, HF patients categorized as New York Heart Association (NYHA) Class III-IV tend to have lower dietary iron intake compared to those in Class II, suggesting a decline in iron intake as HF severity increases [10]. Iron absorption is strongly tied to its bioavailability, which depends on the type of iron consumed, whether haeme or non-haeme [1, 20, 23]. Haeme iron is generally better absorbed, while calcium has been found to inhibit the absorption of both types of iron [20, 23]. Conversely, vitamin C and meat consumption have been associated with increased iron absorption, highlighting the impact of dietary habits on iron absorption [10, 24].

Moreover, genetic mutations, especially in the TMPRSS6 gene, have been associated with the development of iron deficiency in HF [20, 25]. The TMPRSS6 gene encodes a protein called matriptase-2, which plays a crucial role in regulating iron homeostasis by controlling the production of hepcidin, a hormone that inhibits iron absorption and release. Mutations in the TMPRSS6 gene can lead to reduced activity of matriptase-2, causing elevated levels of hepcidin which subsequently impede the absorption of dietary iron [26].

### 2.2 Reduced Iron Absorption

Sandek *et al*. found that HF patients exhibit altered intestinal morphology, hindering iron absorption [13]. Reduced blood supply to the intestines, causing bowel ischemia, exacerbates this issue [10]. Rats with IDA showed compensatory mechanisms such as increased cell proliferation, villous dimensions, and mucosal thickness, but these were ineffective in HF due to intestinal morphological changes [27]. In Dahl salt-sensitive HF rats, important genes for iron absorption like Dcyt-b and DMT-1 were lost. HF rats with IDA showed a minimal increase in intestinal HIF-2-alpha compared to IDA-only rats [13].

**Fig. (2):**
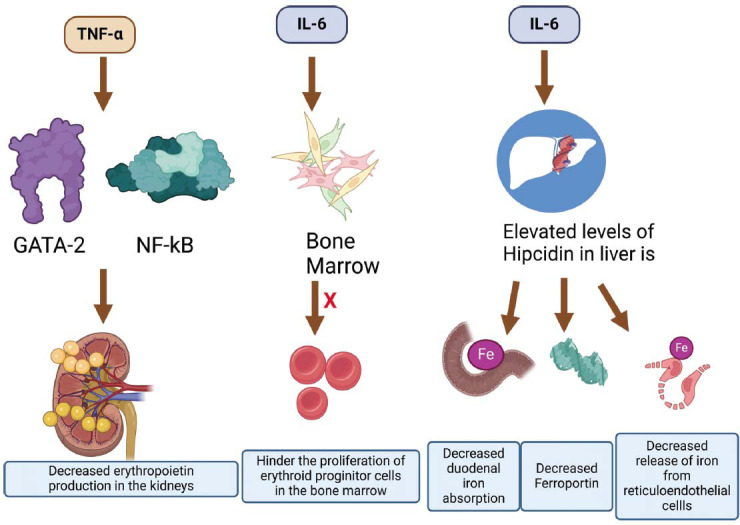
Effect of pro-inflammatory cytokines and hepcidin in the pathophysiology of anemia.

Inflammatory markers like TNF-α and IL-6 rise in HF, activating factors inhibiting erythropoietin. IL-6 and TNF-α indirectly increase hepcidin, which inhibits iron absorption by degrading ferroportin [28, 29] (Fig. **2**). However, contradictory studies exist; some iron-deficient HF patients had lower hepcidin despite higher inflammatory markers. NYHA Class IV HF patients had lower hepcidin levels [10]. A low pH is crucial for iron absorption, while chronic use of Proton Pump Inhibitors and Histamine receptor-2-antagonists decreases oral iron absorption [10, 30].

### 2.3 Role of Erythropoietin

Erythropoietin, responsible for stimulating red blood cell production, is produced by peritubular fibroblasts in the renal cortex and medulla [31, 32]. In HF, damage to this area of erythropoietin production occurs, leading to impaired erythropoiesis [1, 28]. Studies have shown that the Renin-Angiotensin system affects erythropoietin levels; hence, patients on angiotensin-converting enzymes (ACE) inhibitors and angiotensin receptor blockers (ARBs) may experience mildly reduced hemoglobin through two pathways [33, 34]. In addition to decreasing erythropoietin production, these medications block the breakdown of N-acetyl-seryl-aspartyl-lysyl-proline, an inhibitor of hematopoiesis [14, 35].

### 2.4 Myocardial Iron Deficiency

Research indicates various mechanisms leading to myocardial iron deficiency (MID) and systemic iron deficiency [10, 36]. Iron plays essential roles beyond oxygen transport, involving key enzymes in the citric acid cycle and those that degrade reactive oxygen species (ROS) [37, 38]. The study found that HF patients with MID had reduced levels of citric acid cycle enzymes and ROS scavenging enzymes compared to those without MID. This impaired citric acid cycle activity reduces cardiomyocyte metabolic function, affecting maximum exercise capacity in HF with reduced ejection fraction (HFrEF) [16, 39].

Lower mRNA expression of transferrin receptor 1 (TFR1) was observed in HF, leading to increased neuroendocrine system activation, linking TFR1 expression to MID [10, 16]. Additionally, beta receptor agonist-induced HF in mice led to neurohormonal activation and subsequent MID [10].

## 3 DIAGNOSIS OF IRON DEFICIENCY ANEMIA IN HEART FAILURE PATIENTS

**Fig. (3):**
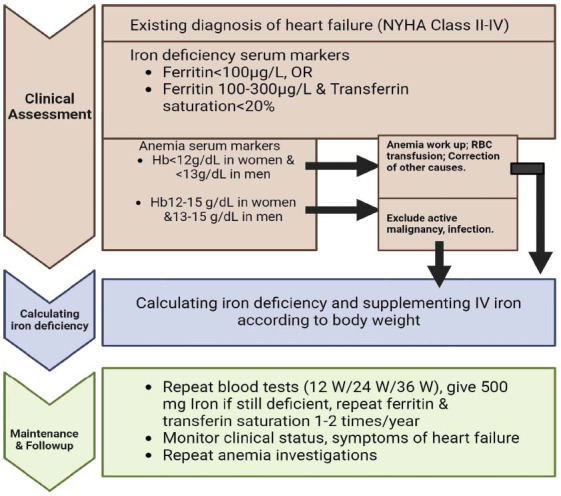
Diagnosing and treating iron deficiency in heart failure.

Multiple criteria might be considered while developing a methodology to diagnose IDA in individuals with HF. Commencing with the initial comprehensive medical history, a physical examination to identify symptoms indicative of HF and anemia and subsequent performance of a complete blood count (CBC) to assess low levels of hemoglobin and hematocrit, which are symptomatic of anemia [40] (Fig. **3**). In addition to observing clinical changes, conducting blood tests, and monitoring overall health deterioration, it is crucial to further investigate the particular diagnosis of IDA in patients with HF [40, 41]. In HF, iron deficiency can be detected by measuring ferritin levels [18, 42]. Iron deficiency is indicated when ferritin levels are below 100 ng/mL or between 100 and 299 ng/mL with a transferrin saturation (TSAT) of less than 20% [43]. It is crucial to ensure that TSAT levels are below 20% since they signal insufficient iron availability to supply cells [44]. Assessing iron deficiency in the bone marrow is feasible and unaffected by inflammation [45, 46]. However, it is constrained by its high expenses, invasive nature, and the need for large-scale implementation [17].

A cohort study involving 42 patients, observed a correlation between TSAT levels below 19.8% and blood iron levels below 13 umol/L with the presence of bone marrow iron insufficiency [47]. In this study, transferrin was used to calculate TSAT rather than total iron-binding capacity.

Additional tests can be taken into account, such as the Perls Stain technique, which uses bone marrow samples to evaluate iron buildup in hemosiderin, hence indicating diminished or missing levels [3]. Another diagnostic test that can be used is measuring the serum iron levels [28]. In cases with inflammatory anemia, these levels are usually low [48]. However, it is important to note that if oral iron treatment is being given, it can falsely appear as high levels [15].

The differentiation between IDA and anemia of chronic disease (ACD) in HF patients is of utmost importance as it might dictate the therapeutic approach and the individual's response to treatment. Patients with anemia with HF commonly exhibit comorbidities such as low albumin levels, cardiovascular issues, and alterations in their hemodynamic status [49]. For example, anemia in cancer patients has been demonstrated to result in dosage reductions, a negative prognosis, and decreased odds of survival due to constraints in hematopoiesis [50].

Diagnosing IDA in patients with HF can be challenging, especially due to factors such as inadequate nutrition and decreased absorption of iron [43]. In some cases of chronic heart failure (CHF), there are alterations in the intestinal wall, specifically involving the malfunctioning of enterocytes, which can exacerbate the problem of reduced iron absorption. In cases of congestive heart failure accompanied by cardiovascular comorbidities, it may be necessary to administer medicines such as anticoagulants and antiplatelet therapy. However, these treatments might worsen chronic blood loss, resulting in decreased iron levels.

## 4 CURRENT RECOMMENDATIONS FOR TREATING IDA AND HF

Due to the association of IDA in HF with morbidity and mortality of the patients, correcting iron deficiency is important to improve the functional status, exercise tolerance, symptoms, and overall quality of life. Treatment for iron deficiency in HF is warranted when ferritin and TSAT levels fall below the aforementioned thresholds, especially in patients with symptomatic HF and reduced ejection fraction (HFrEF) [2]. There are several iron preparations and strategies available to address these issues.

### 4.1 Oral Iron Preparations

The most commonly used oral iron supplements are iron salts, such as ferrous sulfate and ferrous fumarate. Oral iron is known to be more economical than IV iron. A double-blinded randomized controlled trial offered oral ferrous sulfate 200 mg and placebo in a 1:1 ratio for 12 weeks to 54 patients with HFrEF and iron deficiency [51]. After 12 weeks, the study showed a significant improvement in the functional capacity of the patients compared with those who received the placebo. A network meta-analysis of randomized controlled trials compared the efficacy of IV and oral iron for IDA in HF patients [52]. It concluded that oral iron therapy can reduce all-cause death and hospitalizations for HF, though it did not improve exercise capacity and quality of life. Many patients would not prefer oral iron therapy, due to its side effects like constipation, diarrhea, and nausea [53]. However, for many countries, oral iron therapy can be a better alternative due to its availability and favorability to the economy [21, 22].

### 4.2 IV Iron Preparations

Out of the three generations of IV iron preparations, 3rd generation IV preparations such as ferric carboxymaltose (FCM) and ferric derisomaltose (FDI) have shown good safety profiles [51, 54]. 1^st^ generation such as Iron dextran has a higher risk for anaphylaxis, and 2^nd^ generation preparations like iron sucrose can only be given at lower doses due to the oxidative stress [51, 55]. Several trials have been conducted to assess the safety and efficacy of FCM (Table **1**). The HEART-FID trial which compared FCM with a placebo, failed to identify a significant difference in terms of hierarchical composite of death, hospitalizations for heart failure, or 6-minute walk distance [56]. The AFFIRM-AHF trial also showed no apparent change in the risk of cardiovascular death following treatment with FCM but had reduced the risk of HF hospitalization after receiving FCM for IDA while having a <50% LVEF, and were stabilized after an episode of acute HF [57]. This study highlights that the prevalence of iron deficiency is substantial across all HF subtypes, but more work is needed to establish robust evidence for the effectiveness of IV iron therapy in HFpEF. In contrast, the IRONMAN trial which assessed the longer-term effects of intravenous FDI on cardiovascular events in HF patients concluded that FDI administration reduced the risk of hospital admissions for HF and cardiovascular death [58]. The studies which compared oral iron with IV iron have shown that IV iron is superior in improving the hematological values, functional capacity, HF hospitalizations, and the 6-minute walk test [51]. These studies indicate that IV iron is the best available option.

Given the high prevalence of iron deficiency in HFpEF and its potential impact on quality of life, the FAIR-HFpEF trial is investigating the effects of IV iron therapy in HFpEF patients [59]. The trial aims to evaluate improvements in exercise capacity and quality of life, specifically addressing whether the benefits of iron supplementation extend to HFpEF in the same way they do for HFrEF. This trial could lead to more tailored therapeutic strategies for HFpEF patients.

On the other hand, there are potential side effects of IV iron which include mild reactions such as headaches, nausea, and infusion site reactions [60, 61]. However, more serious adverse events like hypersensitivity reactions and anaphylaxis are rare with modern IV formulations like FC or iron isomaltoside [62, 63]. These newer formulations have improved safety profiles compared to older preparations, but concerns about iron overload, particularly in chronic conditions like HF, persist among clinicians [20].

### 4.3 Other Treatment Options

SGLT2 inhibitors such as Dapagliflozin (sodium-glucose co-transporter 2-inhibitor) can increase the hematocrit and hemoglobin levels, suggesting potential use to either correct or prevent anemia in HF patients [64]. DAPA-HF trial (Dapagliflozin and Prevention of Adverse-Outcomes in Heart Failure) allowed us to observe the effect of Dapagliflozin, on iron status and outcomes related to iron status in HFrEF patients. The study confirmed that the HFrEF patients with iron deficiency had worse outcomes compared to the iron-replete patients [26]. Results showed that Dapagliflozin’s effect in iron-deficient patients showed the same outcomes as iron-repleted regarding either worsening HF (hospitalization or urgent visit requiring intravenous therapy) or cardiovascular death. Similarly, no benefit was noted for all-cause mortality. However, it reduced the Transferrin saturation, ferritin, and hepcidin while increasing the TIBC and soluble transferrin receptors indicating an increased iron use. The study concluded that even though Dapagliflozin increases iron use, it will improve HF outcomes irrespective of iron status at baseline.

### 4.4 Dosing Regimens and the Need for Dose Optimization of IV Iron

According to the 2021 ECS guidelines, to improve the symptoms, exercise capacity and the quality of life in patients with LVEF <45% and to reduce the HF rehospitalization in patients with <50% who recently discharged for an acute episode of HF, FCM should be considered [65]. But in the 2023 focused update of the same guidelines, IV iron was recommended for iron-deficient patients with HFrEF or HEmrEF to improve symptoms and quality of life and suggested to consider FCM or FDI to reduce the risk of HF hospitalizations [19]. Another retrospective study discussed the need for dose optimization for patients with high body weight or low hemoglobin when the treatment with FCM was limited to 1 g during one administration [66]. The study analyzed a total of 211 patients and compared the actual given dose *versus* the calculated target and the difference was identified as dose deficit. The study also assessed the impact of the dose deficits after 12 weeks about the clinical and biochemical status. The findings indicated that the majority of patients required a dose exceeding 1g of FCM, and follow-up treatments were suggested to address any residual dosage deficits. Failure to correct these deficits may have led to less improvement in functional and biochemical status.

### 4.5 Iron Supplementation in HF Patients with Chronic Kidney Disease (CKD)

CKD has a well-known association with HF [67]. CKD can cause normocytic anemia and IDA due to different mechanisms [68]. In contrast to IDA in CKD, IDA in HF will cause decreased hepcidin, showcasing the distinct difference between IDA in HF and IDA in CKD-associated HF [30]. In CKD, erythropoiesis-stimulating agents (ESAs) have been shown to improve normocytic anemia but have failed to reduce HF hospitalizations [29]. To improve anemia and outcomes of HF in CKD effects of IV iron were tested. FIND-CKD randomized trial showed that IV iron reached the target ferritin levels quickly and maintained longer than oral iron in CKD patients, while also delaying and/ or reducing the need for ESAs [69]. Additionally, the study evaluated the IV iron dosage required for patients with CKD undergoing hemodialysis. The study analyzing HF events in the PIVOTAL trial concluded that administering high-dose IV iron reduced the risk of HF events in patients undergoing hemodialysis compared to low-dose IV iron [70]. But this became concerning as long-term high dose iron loading can cause oxidative stress, suggesting the need for long-term studies. Meanwhile, novel oral iron agents like ferric citrate have shown promising effects on dialysis-dependent patients, opening up more possibilities to explore more options for IDA in HF patients with CKD [68].

## 5 CLINICAL OUTCOMES AND PROGNOSTIC IMPLICATIONS

IDA affects approximately 40-70% of patients with HF and is considered a negative prognostic indicator [77, 78]. It is well-established that iron deficiency leads to oxidative metabolism, resulting in myocardial cell damage. This adverse condition in the myocardium contributes to cardiovascular dysfunction and manifests clinically as decreased exercise capacity, diminished quality of life, and reduced life expectancy, along with increased cardiovascular morbidity and recurrent hospitalizations [69, 77, 78]. Given the risks associated with iron deficiency in heart failure patients, it is imperative to address this laboratory abnormality. According to guidelines from the European Society of Cardiology (ESC), the American College of Cardiology (ACC), and the American Heart Association (AHA), iron deficiency in HF patients should be treated (level of evidence II A) to improve symptoms, exercise capacity, and quality of life [75, 77]. Treatment of iron deficiency has been associated with decreases in emergency visits, HF readmissions, and all-cause mortality [75, 79]. Contrary to this, HF patients with untreated iron deficiency tend to have a worse prognosis.

A systematic meta-analysis study comparing IV iron treatment with placebo in patients with ID in HF was reviewed [77]. The research encompassed 9 multicenter studies involving a total of 261 patients. It showed a significant reduction in the number of HF hospitalizations and improvement in the NYHA class compared with the placebo. However, there was no significant change in terms of mortality.

Additionally, a Bayesian meta-analysis revealed that IV iron replacement for ID in HF patients reduced the percentage of hospitalizations and cardiovascular mortality [75]. Garg *et al*., in a double-blind study with 3065 patients with HF and ID, compared IV FCM (1532 patients) with placebo (1533 patients) [76]. The results revealed no statistically significant differences in mortality rates (8.6% *vs* 10.3%) or hospitalization rates (31.0% *vs* 33.2%), respectively. Only a modest change was observed in the 6-minute walk distance test.

On the other hand, Kalra *et al*. conducted a comparative study with 1137 patients with HF and ID anemia, dividing them into 2 groups: one group received FDI, and the second group received only usual care [58]. The first group had 250 hospital admissions and 119 deaths, while the second group experienced 313 admissions and 138 deaths. Although the numbers were lower in the first group, statistical analysis revealed no significant difference between the two groups.

Furthermore, an observational retrospective analysis by López-Vilella *et al*. enrolled 890 patients with ID and acute HF with preserved and reduced left ventricular ejection fraction (LVEF), using FCM [80]. It decreased the number of hospitalizations and the percentage of cardiovascular deaths in both groups.

To evaluate the benefits of IV iron medication in patients with iron deficiency and HF on clinical outcomes and mortality, a multicentre double-blind, placebo-controlled trial (CONFIRM-HF) enrolled 301 patients [81]. The study demonstrated that patients treated with FCM showed significant improvement in the 6-minute walk distance test at week 24, decreased incidence of hospital admissions due to HF, and improvement in clinical symptoms and quality of life. Similar results were reported in the double-blind randomized FAIR-HF trial, where FCM medication improved clinical symptoms and the 6-minute walking distance test [69].

According to the AFFIRM-AHF trial, only IV iron replacement improved clinical outcomes and reduced the number of hospitalizations in patients with HF and iron deficiency, contrasting with patients receiving oral iron treatment, who showed no significant improvement [82]. Nonetheless, a non-randomized, open-label study found that oral sucrosomial iron improved exercise capacity and quality of life.

Study cohorts have demonstrated that heart failure patients with IDA, who do not receive iron medication, have an increased risk of mortality and morbidity compared to those who receive IV iron treatment. A retrospective comparative study conducted in Germany, aimed to assess the clinical outcomes of HF patients with IDA who received iron treatment compared to those who did not [78]. The research examined 9,876 patients in total. Results showed that those who didn't receive iron therapy had a higher death rate of 23.6%, compared to 18.5% for patients given IV iron. Additionally, the IV iron group experienced better outcomes, including improved survival rates and fewer, shorter hospital stays, when compared to the untreated group. Thus, treating iron deficiency in HF patients is crucial for improving prognosis, including clinical outcomes, quality of life, hospital admissions, and survival rates.

## 6 BARRIERS TO CLINICAL USE

Despite the compelling evidence, the uptake of iron therapy in HF remains suboptimal. Several barriers contribute to this underuse:

### 6.1 Lack of Familiarity with Guidelines

Many clinicians may not be fully aware of the specific recommendations for iron deficiency management in HF [43]. Additionally, some clinicians may perceive iron deficiency as a secondary issue, rather than a critical therapeutic target in HF [83].

### 6.2 Concerns about Safety

Although adverse events related to IV iron are rare, concerns about hypersensitivity reactions and iron overload can still deter its use [84]. This is particularly true in HF, where physicians may be cautious about adding another treatment to an already complex medication regimen.

### 6.3 Cost and Accessibility

The cost of IV iron preparations, particularly in settings without insurance coverage, can be a barrier to treatment [85]. Oral iron is inexpensive and widely available, but its limitations in HF patients make it an ineffective option, particularly in severe cases of iron deficiency [41].

### 6.4 Weak Guidelines

While the ESC and ACC both support the use of IV iron in HF patients, the recommendations are not as strongly emphasized as other HF therapies like beta-blockers or renin-angiotensin system inhibitors [86-88]. This contributes to a perception that treating iron deficiency is not as critical to patient outcomes. Stronger recommendations might encourage more widespread use, especially if future large-scale trials provide even more definitive evidence.

## 7 CHALLENGES AND FUTURE DIRECTIONS

One of the key challenges in treating IDA in HF patients is compliance with oral iron therapy. Oral iron is inexpensive and widely available, but gastrointestinal side effects such as nausea, constipation, and discomfort, which affect up to 70% of patients, often lead to poor adherence [89]. This limits its effectiveness, especially for long-term management in HF patients who require sustained iron repletion. Future strategies to improve patient adherence to oral iron therapy are needed, including better formulations or side-effect management.

Absorption of oral iron is another challenge, particularly in patients with HF [90, 91]. Inhibitors of iron absorption, such as calcium-rich foods, tea, and coffee, can further reduce the efficacy of treatment [92]. Despite recommendations to take iron supplements with vitamin C to improve absorption, the complex dietary adjustments required may further hinder compliance [93, 94]. Additionally, conditions associated with HF, such as chronic inflammation and intestinal edema, may impair iron absorption even in ideal circumstances [95, 96].

While IV iron supplementation is a more effective treatment for those with severe iron deficiency or intolerance to oral iron, its cost and availability can be barriers, particularly in resource-limited settings [97-99]. The higher price and need for clinical administration make IV iron less accessible, which may delay treatment for patients who could benefit from rapid iron repletion [100, 101].

Most clinical trials, including those assessing the benefits of iron supplementation, have focused on patients with HF with reduced ejection fraction (HFrEF). There is still limited data on HF with preserved ejection fraction (HFpEF), despite the prevalence of iron deficiency in this group. Future research must explore the effectiveness of iron supplementation in HFpEF to provide more robust evidence for this subgroup.

Future research should aim at developing oral iron supplements with fewer gastrointestinal side effects and better bioavailability. Modified-release formulations or combining iron with agents that enhance absorption could improve patient adherence and the overall effectiveness of oral iron therapy in HF patients. Innovations in oral iron supplements, such as those with enhanced tolerability, could address the significant compliance challenges seen in current treatments.

A more personalized approach to treating iron deficiency in HF patients may improve outcomes. Future studies could identify biomarkers or genetic factors that predict individual responses to oral or parenteral iron therapy. Tailoring treatment based on the patient's specific needs, such as absorption capability, iron metabolism, and heart failure subtype, could enhance treatment efficacy and reduce side effects.

Although IV iron has demonstrated effectiveness in HFrEF, ongoing and future trials like FAIR-HFpEF will determine its benefit in HFpEF. These results could expand the use of IV iron in HFpEF patients and guide treatment protocols for this less-studied subgroup. In addition, evaluating the long-term benefits of IV iron on clinical outcomes such as mortality, hospitalization rates, and quality of life in a broader spectrum of HF patients is crucial.

Efforts to reduce the cost of IV iron formulations, improve healthcare policies, and increase accessibility in low-resource settings are essential for the broader adoption of this therapy. Health systems may need to explore cost-effective ways of delivering IV iron, including outpatient infusion clinics, mobile healthcare units, or home-based IV iron administration for certain patients.

Future research should investigate the efficacy of combining oral and parenteral iron therapy or adjunct therapies (such as anti-inflammatory agents) to optimize iron status in HF patients. Understanding how other treatments may interact with iron supplementation in managing HF is crucial to improving overall patient care.

## CONCLUSION

Iron deficiency anemia in patients with heart failure is associated with a worsened prognosis and severe symptoms. Studies on iron deficiency anemia in heart failure patients have demonstrated decreased exercise capacity, heightened cardiac morbidity, and mortality. Screening and treating iron deficiency anemia are crucial to prevent exacerbation of heart failure symptoms. Numerous studies indicate that treating iron deficiency anemia may improve overall quality of life. Diagnosis requires assessing ferritin and transferrin saturation levels through blood work. Treatment options include oral iron supplements or parenteral iron therapy. However, conflicting results from studies suggest uncertainty regarding the cardiovascular benefits of treating iron deficiency anemia in heart failure patients. Nevertheless, consensus suggests that treating iron deficiency anemia may mitigate symptoms that worsen heart failure. Intravenous iron replacement therapy has shown superior efficacy compared to oral supplements alone, although the latter is more cost-effective. Moving forward, personalized treatment plans for heart failure patients with concurrent iron deficiency anemia are essential to enhance quality of life and survival rates.

## Figures and Tables

**Table 1:** Some of the studies on treatment options of IDA in HF patients.

**Study**	**Objective**	**Study Population**	**Method**	**Outcomes**
Jankowska *et al*. [12]	To evaluate the impact of IV ferric carboxymaltose (FCM) *vs*. placebo on HRQoL for the AFFIRM-AHF population.	1058 patients (535 FCM and 523 placebo) who completed the baseline 12-item Kansas City Cardiomyopathy Questionnaire (KCCQ12) from the AFFIRM-AHF study population as above.	KCCQ-12 was assessed at weeks 2,4,6,12,24,36 and 52. The overall summary score (OSS) and clinical summary score (CSS) were compared which were extracted from KCCQ-12.	Treatment with FCM resulted significant benefits on HRQoL in the patients with iron deficiency and LVEF an acute HF episode compared to the placebo. The beneficial effects resulted starting from the 4th week of treatment and it lasted through week 24.
Cleland *et al*. [13]	To identify the relationship between blood tests for iron deficiency and anemia, with the response of HF patients to IV iron therapy.	1137 HF patients with LVEF is 45% or less, and either serum ferritin < 100 µg/L or transferrin saturation (TSAT) < 20%.	Patients were randomized to receive either ferric derisomaltose (FDI) or usual care. Then they compared baseline anemia severity, ferritin and TSAT, hemoglobin change from baseline, 6 MWDT, and Minnesota living with HF (MLwHF) score at 4 months along with Clinical events such as HF hospitalization or cardiovascular death.	Identifying patients who will benefit most from IV iron treatment may be possible by assessing the level of anemia or TSAT, particularly in patients with HF. However, this interpretation may require confirmation.
Suryani *et al*. [52]	To evaluate the effect of oral iron in the form of ferrous sulfate on functional capacity in HF patients with IDA.	54 patients were considered for the trial who had HFrEf <45% with NYHA class II-III and IDA hemoglobin (Hb) <13 g/dl for men and <12 g/dl for women, ferritin<100 ng/ml or if between 100-300 ng/ml with transferrin saturation <20%.	The study population was randomized 1:1 to receive either oral ferrous sulfate (FS) 200 mg thrice a day or a placebo for 12 weeks. The primary outcome was the response to the 6-minute walking test.	12 weeks of oral ferrous sulphate significantly improved functional capacity and NYHA functional class in HFrEF patients with IDA. And oral FS can be easily considered as an alternative in developing countries.
Ponikowski *et al*. [57] (AFFIRM-AHF Trial)	To assess the outcomes of patients who received ferric carboxymaltose and were stabilized after an acute heart failure episode, compared to the effects of a placebo.	After screening 1525 patients, 1132 were assigned. They were either age 18 or older, with an acute HF hospitalization, and have iron deficiency: ferritin level <100 µg/L or if 100-299 µg/L have a transferrin saturation <20%, LVEF at <50%.	Assigned to a 1:1 ratio, before patients get discharged from the hospital to receive either ferric carboxymaltose or placebo for 24 weeks. The dose was decided on the extent of the iron deficiency. Primary outcome- Total HF hospitalizations and cardiovascular death up to 52 weeks from the randomization. Secondary outcome evaluation is done following aspects up to 52 weeks from the point of randomization. •Total HF hospitalizations and Cardiovascular death •Cardiovascular death •Total HF hospitalization •Time to first HF hospitalization or cardiovascular death •Days lost due to the HF hospitalizations and cardiovascular death.	FCM was safe and reduced the HF hospitalizations in iron deficient, HF patients with LVEF acute episode of HF. The study did not show a significant effect on the risk of CV deaths.
Jhund *et al*. [67] (The Proactive IV Iron Therapy in Hemodialysis Patients) PIVOTAL trial.	To assess the impact of intravenous iron on heart failure events in hemodialysis patients.	2,141 patients who receive maintenance hemodialysis for end-stage kidney disease (ESKD). The therapy was initiated no more than 12 months, with a ferritin level <400 µg/L and transferrin saturation (TSAT) <30%. These patients were receiving Erythropoiesis stimulating agent (ESA) when enrolled.	Compared the high-dose iron therapy in the form of iron sucrose with low-dose iron therapy in hemodialysis patients.12 Heart failure hospitalization was a decided outcome, and this study has analyzed the HF event of the trial. In the PIVOTAL trial, the participants stopped any existing iron treatments and were randomly assigned in a 1:1 ratio to receive either high-dose (400 mg of iron sucrose) proactively or low dose (0-400 mg of iron sucrose monthly) of IV iron reactively. Overall, 2,141 participants were followed for a median of 2.1 years.	The high-dose IV regimen decrease the risk of first and recurrent HF events in hemodialysis patients, compared to the low-dose regimen.
Macdougall *et al*. [69]	To assess if the coexisting kidney impairment affects the efficacy and the safety of ferric carboxymaltose.	The study population of the AFFIRM-AHF. The study excluded dialysis patients.	Same as AFFIRM-AHF and the stratified group, according to the baseline eGFR was analyzed in this sub-group analysis.	The efficacy and the safety of ferric carboxymaltose didn’t get affected by the kidney functions and therefore for the management of the iron deficiency in patients with reduced ejection fraction. FCM can be used regardless of kidney function.
Docherty *et al*. [64]	To analyze the effects of dapagliflozin in HF, according to the iron status at baseline.	4744 patients were recruited to the DAPAHF according to the NYHA class II-IV, LVEF ≤ 40% and an elevated NT-proBNP (N-terminal pro-B-type natriuretic peptide) level, who have been optimally treated according to local guidelines. Out of these patients, 3009 had the measurements for ferritin and transferrin at baseline and 1314 were deficient in iron.	The study compared the efficacy and the safety of dapagliflozin 10mg once daily with a placebo when added to the standard care of HFrEF. The primary outcome was HF events including hospitalization or episodes needing IV medication or cardiovascular death. In the subgroup analysis they have defined iron deficiency as ferritin level level is 100 to 299 ng/mL, transferrin saturation.	Dapagliflozin can improve the HF outcomes despite the iron status at baseline.
Lewis *et al*. [71] (IRONOUT-HF)	To test if oral iron polysaccharide improves peak exercise capacity in patients with Heart Failure with reduced Ejection Fraction (HFrEF) and iron deficiency.	225 patients with HFrEF (<40%) and iron deficiency with serum ferritin level of 15 to 100 ng/mL or if serum ferritin level of 101 to 299 ng/mL with transferrin saturation of <20%.	Patients were randomized 1:1 to receive oral iron polysaccharide 150 mg twice daily for 16 weeks or placebo. The change in peak oxygen uptake (V̇ O2) from baseline to 16 weeks is considered the primary endpoint. Secondary endpoints were •Change in 6 min walk distance •Plasma N-terminal pro-B type natriuretic peptide (NT-proBNP) levels •Health status according to the Kansas City Cardiomyopathy Questionnaire	High dose oral iron polysaccharide did not improve the exercise capacity (determined by peak oxygen uptake (V̇ O2), 6min walking distance, NT-pro BNP and status according to Kansas City Cardiomyopathy Questionnaire) in the iron deficient patients with HFrEF compared to the placebo.
Okonko *et al*. [72] (FERRIC-HF)	To check the hypothesis of IV iron improves exercise tolerance in both anemic and non-anemic iron-deficient patients with symptomatic heart failure.	35 patients with chronic heart failure in the age range of 64 ± 13 years with peak oxygen consumption (pVO2) 14.0 ± 2.7 ml/kg/min. Ferritin level was <100 ng/ml and if ferritin was 100-300 ng/ml, transferrin had to be <20%. The anemic group had hemoglobin <12.5 g/dl versus nonanemic group with 12.5-14.5 g/dl.	The study employed a randomization ratio of 2:1, where the study group received 16 weeks of IV iron in the form of iron sucrose: 200 mg weekly, with a reduction to 200 mg monthly once ferritin levels reached >500 ng/ml, while the control group received no treatment. The primary endpoint was the change in absolute pVO2.	IV iron can improve the exercise capacity and the symptoms of the HF patients with iron deficiency and anemic patients showed to be more benefitted.
Gutzwiller *et al*. [73] (FAIR-HF sub-analysis)	To evaluate the HF patients with iron deficiency who are receiving either IV iron or Placebo in terms of determinants of health-related quality of life (HRQoL).	Same as FAIR-HF.	As a sub-analysis of FAIR-HF, this multivariate analysis was conducted on clinical variables, as independent variables and HRQoL measurements as dependent variables.	The measures of HRQoL were influenced IV Iron treatment, NYHA class, history of stroke, renal function, exercise tolerance and country of residence in HF patients with iron deficiency. So, interventions to improve exercise tolerance, stroke prevention and improve NYHA class in the clinical setup will help the outcomes of HF patients with iron deficiency and receive IV iron. Also, it is advisable to closely monitor renal functions and iron status for better outcomes. This is overall important to identify the patients who are at higher risk for reduced HRQoL and to intervene appropriately.
Filippatos *et al*. [74]	To investigate if the beneficial outcomes of IV iron are independent of anemia in patients with chronic heart failure (CHF).	The study population of the FAIR-HF trail.	From the data collected in the FAIR-HF trial, the study analyzed the efficacy and the safety of the IV iron according to the presence or absence of anemia at baseline. Anemia was considered as Hb ≤ 120 g/L.	FCM treatment for iron deficiency in CHF is effective and safe despite the anemic status. Therefore, assessing the iron status of symptomatic CHF patients and treatment with FCM will be beneficial.
Anker *et al*. [75]	To evaluate the precise estimates of the effect of IV iron on recurrent HF hospitalizations and cardiovascular mortality using consistent subgroups across trials in iron-deficient patients with HFrEF.	Study population used in FAIR-HF (n = 459), CONFIRM-HF (n = 304), AFFIRMAHF (n = 1108), and IRONMAN (n = 1137) trials.	Individual data from FAIRHF, CONFIRM_HF, and AFFIRM_HF trials were reanalyzed to align closely with the approach of the IRONMAN trial. Definitions and subgroups from the above three trials were matched to IRONMAN. Primary end point-recurrent HF hospitalizations and cardiovascular mortality. They used the rate ratios (RR) from the Lin-WeiYang-Ying model to analyze the recurrent HF admissions and the Bayesian random effects meta-analysis to pool the data.	IV Iron significantly reduces the recurrent HF hospitalizations and cardiovascular mortality in HFrEF patients with iron deficiency and results were consistent in the subgroups studied.
Mentz *et al*. [76] (HEART-FID)	To assess the effect of FCM, on the incidence of death and HF hospitalization and 6 minutes walk distance compared to the placebo in iron-deficient patients with HFrEF.	3065 ambulatory patients with LVEF 40% or less and with iron deficiency.	The study assigned patients in a 1:1 ratio to either receive IV FCM or placebo in addition to the standard therapy for HF.	With respect to the hierarchical composite of death, hospitalizations for heart failure, or 6- minute walk distance, Ferric carboxymaltose failed to show an apparent difference compared to the placebo in ambulatory patients with iron deficiency and HFrEF.

**Abbreviations used in the Table**: IV- Intrevenou; HRQoL- Health related quality of life; FCM -Ferric carboxymaltose; KCCCQ-12 -12-item Kansas City Cardiomyopathy Questionnaire; OSS- Overall summary score; CSS- Clinical summary score; LVEF -Left ventricular ejection fraction; HF -Heart failure; TSAT-Transferrin saturation; FDI -Ferric derisomaltose; Hb -Hemoglobin;6MWT- 6min walking distance test ; MLwHF- Minnesota living with heart failure;IDA- Iron deficiency anemia; HFrEF- Heart failure with reduced ejection fraction; NYHA- New York Heart Association; FS- Ferrous sulfate; CV-Cardiovascular; PIVOTAL trial -The Proactive IV Iron Therapy in Hemodialysis Patients; ESKD-End stage kidney disease ;ESA- Erythropoiesis stimulating agent; eGFR- estimated glomerular filtration rate; pV̇O 2 -Peak oxygen uptake; NT-proBNP- Plasma N-terminal pro-B type natriuretic peptide; CHF -Chronic heart failure.

## LIST OF ABBREVIATIONS

ACC: American College of Cardiology
ACE: Angiotensin-converting Enzymes
AHA: American Heart Association
ARBs: Angiotensin Receptor Blockers
CHF: Chronic Heart Failure
ESAs: Erythropoiesis-stimulating Agents
ESC: European Society of Cardiology
HF: Heart Failure
IDA: Iron Deficiency Anemia
IV: Intravenous
